# Intestinal parasitic infections in relation to CD4^+^ T cell counts and diarrhea in HIV/AIDS patients with or without antiretroviral therapy in Cameroon

**DOI:** 10.1186/s12879-016-1337-1

**Published:** 2016-01-11

**Authors:** Dickson Shey Nsagha, Anna Longdoh Njunda, Nguedia Jules Clement Assob, Charlotte Wenze Ayima, Elvis Asangbeng Tanue, Odette Dzemo kibu, Tebit Emmanuel Kwenti

**Affiliations:** 1 Department of Public Health and Hygiene, Faculty of Health Sciences, University of Buea, Box 63, Buea, Cameroon; 2 Department of Medical Laboratory Sciences, Faculty of Health Sciences, University of Buea, Buea, Cameroon

**Keywords:** HIV/AIDS, Intestinal parasitic infection, CD4^+^ T cell count, Diarrhea, Antiretroviral therapy, Cameroon

## Abstract

**Background:**

Intestinal parasitic infections (IPI) are a major public health concern in HIV/AIDS patients particularly in resource-limited settings of Sub-Saharan Africa. Studies investigating the relationship between intestinal parasitic infections and CD4^+^ T cell counts and diarrhea in HIV/AIDS patients with or without antiretroviral therapy in the region are not readily available hence the need to perform this study.

**Methods:**

In a comparative cross-sectional study involving 52 pre-ART and 248 on-ART HIV patients. Stool samples were collected and analysed for intestinal parasites by wet and iodine mounts, Kato-Katz, formol ether, modified field staining, and modified Ziehl-Neelsen staining techniques. Blood samples were collected and analysed for CD4^+^ T cell counts by flow cytometry. A pre-tested semi-structured questionnaire was used to collect data on socio-demographic and clinical presentation. Data were analysed using STATA version 12.1. Statistical tests performed included the Pearson Chi-square, logistic regression and student’s *t*-test. *P* < 0.05 was considered to be statistically significant.

**Results:**

The prevalence of intestinal parasitic infections in pre-ART and on-ART was 84.6 % and 82.3 % respectively with no significant difference observed with respect to age (*p* = 0.06), and gender (*p* = 0.736). All the opportunistic parasites including *Cryptosporidium parvum*, *Cyclospora cayetanensis*, *Isospora belli* and *Microsporidium spp*. were isolated from both groups, with only *Microsporidium spp*. significantly associated with CD4^+^ T cell counts below 200 cells/μl in pre-ART (*p* = 0.006) while *Cryptosporidium parvum*, *Microsporidium spp*. and *Isospora belli* were associated with counts below 200 cells/μl in on-ART. *Cryptosporidium parvum* was significantly associated with diarrhea in pre-ART (*p* = 0.025) meanwhile it was significantly associated with diarrhea in on-ART (*p* = 0.057). The risk of diarrhea was highest in patients with CD4^+^ T cell counts below 200 cells/μl (COR = 10.21, *p* = 0.000) for both pre- and on-ART treatment.

**Conclusion:**

A very high prevalence of intestinal parasitic infections was observed, which did not differ with respect to ART status. All known opportunistic parasites were isolated in both pre-ART and on-ART patients. Low CD4^+^ T cell count may appear to be a factor for intestinal parasitic infections and development of diarrhea. Regular screening and treatment of intestinal parasitic infections is very vital in improving the overall quality of care of HIV/AIDS patients.

## Background

The neglected intestinal parasitic infections (IPI) which are caused by either helminthes or protozoa or both are among the most prevalent parasitic infections responsible for significant morbidity particularly in resource limited settings of developing countries. The incidence of IPI is approximately 50 % in developed countries whereas it reaches up to 95 % in developing countries, with Sub-Saharan Africa having the highest burden [1–3]. The Sub-Saharan African region also has the highest burden of Human Immunodeficiency Virus (HIV) with an estimated 23.5 million cases [4]. With the overlapping distribution of these infections, concomitant infection between HIV and one or more intestinal parasites are therefore common. Although helminthes have co-existed with humans for a long time and have probably adapted for long-term survival in the human host causing relatively minor pathology, HIV is relatively a newcomer that destroys the immune defense of the host, making it vulnerable to other pathogens [5].

Factors prompting transmission of IPI and HIV are most common in the developing countries [6]. In Cameroon like most developing countries in Sub-Saharan Africa, intestinal parasites are widely distributed partly due to the low level of environmental and personal hygiene, faecal contamination of food and drinking water and poor housing. An estimated 80 % of Acquired Immune Deficiency Syndrome (AIDS) patients die of AIDS-related opportunistic infections rather than from the HIV virus itself [7, 8]. Opportunistic parasites such as *Cryptosporidium parvum*, *Cyclospora cayetanensis*, *Isospora belli*, and *Microsporidia spp*. are a common feature in HIV/AIDS persons especially when the CD4^+^ T cell counts fall below 200 cells/μl [7–9].

Studies investigating the association between intestinal parasites and the immune status of HIV patients have reported conflicting outcomes. Some studies have reported a higher prevalence of IPI in patients with CD4^+^ T cell counts below 200 cells/μl [10, 11]. Other studies have reported an increased risk of helminthes infection in HIV patients with higher CD4^+^ T cell count [12, 13]. Meanwhile others have failed to observe any significant association [14–17]. Just a few studies published currently have actually investigated the effect of antiretroviral therapy in individuals with concomitant infection with intestinal parasites and HIV, with some of these studies suggesting that antiretroviral therapy may have a role to play in the risk and severity of intestinal parasites in HIV/AIDS patients [18, 19].

Various microorganisms have been isolated from HIV-infected patients with chronic diarrhea either singly or in combinations [20]. Diarrhea remains a leading cause of morbidity and mortality in the developing world and accounts for over 50 million deaths globally [21] and has been ranked third among diseases responsible for human mortality in the world [22]. It occurs in 30 to 60 percent of North American and European patients and almost 90 % of AIDS patients in developing countries [23] including Cameroon.

In the present study, we determined the relationship between IPI and the CD4^+^ T cell count as well as the diarrheic status of HIV infected persons who were on antiretroviral therapy compared to those who were not on antiretroviral therapy.

## Methods

### Study area

**Fig. 1:**
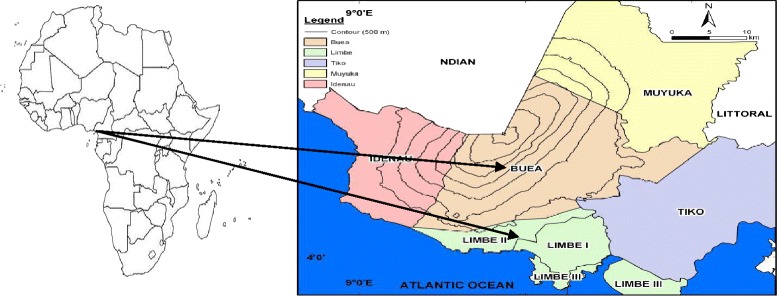
Map of Africa showing Cameroon and the two study sites. Source: Orock FT and Lambi CM, 2014 [25]. Permission was granted by the copy-right holders to reproduce this figure

Participants were enrolled in the HIV treatment centers of the Buea and Limbe Regional Hospitals located in Buea and Limbe of Cameroon. Buea (4°10′N 9°14′E) has a population of about 200,000 inhabitants and Limbe (4°01′N 9°13′E) has a population of about 151,258 inhabitants are the two major urban centers in the Fako division of the South West Region of Cameroon. These two hospitals are the only HIV treatment centers in Fako Division serving the entire population. Limbe is the head quarter of the Cameroon Development Corporation (CDC), the second largest employer in Cameroon after the government and also a major tourist sites with features such as the beach, botanical garden and the zoo. The Mountain in Buea is also a major tourist site and Buea is home to the first Anglo-Saxon state university; the University of Buea. Almost all ethnic groups in Cameroon are represented in these towns attracted by the business friendly environment and job opportunities. The 8 % HIV of the South West Region is one of the highest in the country [24]. Figure 1 [25] shows the two study communities in the South West Region of Cameroon.

### Study design and Setting

This was a hospital-based cross-sectional study in which participants of the Buea and Limbe Regional Hospitals HIV treatment centers were enrolled between April and July 2014.

### Sample size calculation

The sample size was determined using the formula for sample size calculation [26].

$$ \mathrm{n}=\frac{{\left(\mathrm{Z}\right)}^2\mathrm{p}\left(1-\mathrm{p}\right)}{{\mathrm{e}}^2} $$

Where n = sample size,

$$ \mathrm{Z} = \mathrm{constant} = 1.96 $$

*p* = prevalence of intestinal parasites among HIV positive patients = 27.9 % [27].

e = precision of the event of interest = 0.05

$$ \mathrm{n}=\frac{(1.96)^2(0.279)(0.721)}{0.05^2}\kern0.75em  = 309\ \mathrm{participants} $$

A total of 313 patients were contacted but only 300 were consecutively recruited during the study period.

### Sampling technique

A time limited sampling technique was used, where patients were consecutively recruited into the study. Participants were enrolled into the study provided they gave their consent and met the inclusion criteria.

### Study population

HIV patients attending the HIV treatment centers of the Regional Hospitals in Buea and Limbe were approached to take part in the study. The study population was made up of HIV patients who had not yet commence treatment (pre-ART) and HIV patients who were already receiving treatment (on-ART). To be included in the study, patients irrespective of their ages and gender were eligible to participate. Individuals that were on any anti-parasitic medication prior to data collection were excluded from the study.

### Data collection and processing

#### Administration of Questionnaire

A pretested semi-structured questionnaire was administered in order to collect socio-demographic characteristics and clinical information from consented participants who agreed to participate in the study.

### Specimen collection

Stool samples were collected into wide open-necked, sterile containers which were provided to the participants. The participants were instructed on how to collect stool samples at the point of care. These samples were analysed within one hour of collection for ova, cyst and parasites. Samples were stored in 10 % formol ether for 24 h and later stained for intestinal oocyts. After the provision of stool samples, about 4 ml of peripheral blood was collected into EDTA test tubes. These samples were immediately used for the analysis of the CD4^+^ T cell counts.

### Parasitological analysis of stool specimen

The presence of diarrhea was defined as a history of frequent passage (3 times or more) of watery stool in a 24 h period and confirmed by macroscopic examination of stool sample. Stool samples were analysed using saline and iodine mount, Kato-katz [28] and formol ether concentration techniques [29]. Smears prepared from the formol ether concentration techniques were stained using the Modified field staining technique for the detection of spores of *Microsporidium* species [30], and the Modified Ziehl-Neelsen staining technique was used for the detection of oocysts of *Cryptosporidium* species, *Isospora belli*, and *Cyclospora cayetanensis* [31].

### Analysis of CD4^+^ T cell count

CD4^+^ T cell counts were determined using the BD FACSCount™ System (BD Biosciences, USA) as per the manufacturer’s instructions. Briefly, CD4 reagent tubes were labeled and vortexed upside down for 5 seconds and upright for 5 seconds. The reagent tubes were opened with the coring station. EDTA tubes containing blood were inverted 5 to 10 times to adequately mix the blood. 50 μl of whole blood was pipetted into the reagent tubes. Each tube was caped and vortexed upright for 5 seconds. The tubes were incubated for 60 to 120 min at room temperature in the dark. The tubes were uncapped and 50 μl of fixative solution were pipetted into the tubes. The tubes were recapped and vortexed for 5 seconds.

The samples were vortexed upright for 5 seconds before they were analysed on the FACS Count instrument [32].

### Ethical considerations

Ethical clearance was obtained from the Institutional Review Board of the Faculty of Health Sciences of the University of Buea (Reference Number: 2014-02-0214). Administrative authorization was obtained from the South West Regional Delegation of the Ministry of Public Health and the District Health Services. The purpose of the study and the role of the participants were well explained verbally and in the consent form to the participants and participation could only take place after the participant had read and signed the informed consent form voluntarily.

### Statistical analysis

Data were analysed using STATA version 12.1. Statistical significant differences in proportions were evaluated by Pearson’s Chi-square test. The student’s *t*-test was used to compare groups mean. Bivariate analysis was used to assess the effects of intestinal parasites on the CD4^+^ T cell counts and diarrheic state of the study participants. Where appropriate the Fisher exact test was used to determine *p*-values. Statistical significance was set at *p* < 0.05.

### Limitations to the study

Molecular identification techniques for parasitic species were not done due to sparsely equipped laboratories. Also Only single stool samples were collected from each participant. However, to overcome these limitations each stool sample was analyzed more than once using different techniques in order not to miss out parasites present in low quantity.

## Results

### Socio-demographic characteristics of the respondents

At the end of the study, 300 participants were successfully enrolled. Among them were 64(21.3 %) males and 236(78.6 %) females. The ages of the participants ranged between 21–65 years with mean (±SD) age, 40(±10) years.

Fifty two (17.3 %) of the 300 participants were pre-ART meanwhile 248 (82.7 %) were on-ART. The mean age of on-ART patients was 42.6(±9.9) years while that of pre-ART patients was 40.2(±10.2) years. No significant difference was observed in the mean ages between the two groups (*p* = 0.06). Among the 52 pre-ART HIV patients, were 40 (76.9 %) females and 12 (23.1 %) males while among the 248 on-ART HIV patients were 196 (79.1 %) females and 52 (20.9 %) males. No significant difference was observed with respect to gender in the two groups (*p* = 0.736).

### Prevalence of intestinal parasite in pre-ART and on-ART HIV/AIDS patients

Intestinal parasites were isolated from 248 (82.6 %) of the 300 participants. Intestinal protozoa were more prevalent than intestinal helminthes [223 (74.3 %) vs. 34 (11.3 %)]. IPI was more prevalent in females [199 (84.3 %)] than in males [49 (76.5 %)] (*p* = 0.14). Overall, the prevalence of IPI was higher in HIV patients above 60 years [13 (92.9 %)] (Table 1). Among pre-ART HIV patients, the prevalence of IPI was highest in the age group 31–40 meanwhile among on-ART HIV patients the prevalence was highest in patients above 60 years. However, no statistical significance was observed between prevalence of IPI and age group (*p* = 0.47).

**Table 1:** Distribution of IPI with age in the study population

Age category	Total number of participants (No)	Pre-ART No (%)	On-ART No (%)	Overall No (%)
21 – 30	47	11(84.6)	27(77.1)	38(80.9)
31 – 40	118	23(92.0)	76(81.7)	99(83.9)
41 – 50	89	10(76.9)	60(77.9)	70(78.7)
51 – 60	32	0(0.0)	28(90.3)	28(87.5)
>61	14	0(0.0)	13(92.9)	13(92.9)
Total	300	44	204	248

The number of pathogens exceeds the number of patients as multiple parasites were identified in some patients

The prevalence of IPI was higher in pre-ART HIV patients [44/52 (84.6 %)] than in on-ART HIV patients [204/248 (82.3 %)]. However no significant association was observed between prevalence of intestinal parasite and ART (*p* = 0.900). Among pre-ART patients, IPI was higher among males [36 (90 %)] than in females [10 (83.3 %)] meanwhile among on-ART HIV patients, IPI was higher among females [163 (83.2 %)] than in males [39 (75 %)]. No significant association was observed in the prevalence of IPI and gender in both pre-ART (*p* = 0.526) and on-ART (*p* = 0.178) HIV patients.

The commonest intestinal parasite was *Cryptosporidium parvum* [132 (44 %)], while the least prevalent was *Strongyloides stercoralis* [2(0.7 %)]. *Cryptosporidium parvum* was also the most prevalent intestinal parasite in both pre-ART and on-ART HIV patients (Table 2). Species-specific analysis revealed that no particular intestinal parasite was statistically significantly associated with ART status (Table 2).

**Table 2:** Species-specific prevalence of intestinal parasites among HIV-infected persons stratified according to ART status

Parasites identified	Overall No (%)	ART Status		*P*-value
		On-ART No (%)	Pre-ART No (%)	
Protozoa				
*C. parvum*	132(44.0)	109 (53.4)	23 (52.3)	0.971
*B. hominis*	75(25.0)	59 (28.9)	16 (36.4)	0.292
*Microsporidiumsp*	64(21.3)	50 (24.5)	14 (31.8)	0.280
*E. hystolytica*	22(7.3)	18 (8.8)	4 (9.0)	0.913
*I.belli*	13(14.3)	12 (5.9)	1 (2.3)	0.349
*C.cayetanensis*	11(3.6)	9 (4.4)	2 (4.6)	1.000*
*G.lamblia*	10(3.3)	8 (3.9)	2 (4.6)	0.686*
Helminths				
*A.lumbricoides*	13(4.3)	12 (5.9)	1 (2.3)	0.349
*Hookworm*	8(2.7)	8 (3.9)	0 (0.0)	-
*T.trichuria*	7(2.3)	6 (2.9)	1 (2.3)	0.830*
*H. nana*	4(1.3)	4 (2.0)	0 (0.0)	-
*S. stercoralis*	2(0.7)	2 (1.0)	0 (0.0)	-
Total	248 (82.6)	204 (82.3)	44 (84.6)	

*P value from Fisher’s exact test

### Association of intestinal parasite with CD4^+^ T cell count among pre-ART and on-ART HIV/AIDS patients

The prevalence of IPI was highest [71 (93.4 %)] in patients with CD4^+^ T cell count below 200 cells/μl, (Table 3). A highly significant association was observed between prevalence of IPI and low CD4^+^ T cell count (*p* = 0.000). Among pre-ART patients, the prevalence of IPI was highest in individuals with CD4^+^ T cell count below 200 cells/μl (*p* = 0.004) meanwhile among on-ART HIV patients, the prevalence was highest in patients with CD4^+^ T cell count between 200–499 cells/μl (*p* = 0.000).

**Table 3:** Prevalence of intestinal parasitic infection with respect to CD4^+^ T cell count

CD4^+^ T cell count	Pre-ART		On-ART		Overall	
	Present No (%)	Absent No (%)	Present No (%)	Absent No (%)	Present No (%)	Absent No (%)
<200cells/μl	17(100)	0(0)	54(91.5)	5(8.5)	71 (93.4)	5 (6.5)
200 – 499cells/μl	20(95.2)	1(4.8)	93(92.1)	8(7.9)	113 (92.6)	9 (7.3)
≥500cells/μl	9(64.3)	5(35.7)	55(62.5)	33(37.5)	64 (62.3)	38 (37.6)
Total	46	6	202	46	248	52
*χ* ^2^, *P*-value	*χ* ^2^ = 11.179, *p* = 0.004		*χ* ^2^ = 34.137, *p* = 0.000		*χ* ^2^ = 42.825, *p* = 0.000	

*χ* ^2^ = Chi square values for the association between CD4^+^ T cell counts and Antiretroviral therapy

Species-specific analysis revealed that *Cryptosporidium parvum*, *Microsporidium spp*., *Isospora belli* and hookworm were the parasite species most significantly associated with low CD4^+^ T cell count (*p* = 0.000, *p* = 0.000, *p* = 0.003 and *p* = 0.034 respectively) (Table 3). Among pre-ART HIV patients, only *Microsporidium spp*. was significantly associated (*p* = 0.006) with low CD4^+^ T cell count meanwhile *C. parvum*, *B. hominis*, *Microsporidium spp*. and *I. belli* were significantly associated with low CD4^+^ T cell count among on-ART HIV patients (*p* = 0.000, *p* = 0.039, *p* = 0.000 and *p* = 0.003 respectively) (Table 4).

**Table 4:** Prevalence of intestinal parasite among HIV/AIDS patients in relation to their CD4^+^ T cell Counts among pre-ART and on-ART HIV patients

Parasite species	No (%)	Pre-ART				On-ART				Overall			
		CD4^+^ T cell counts				CD4^+^ T cell counts				CD4^+^ T cell counts			
		<200 No(%)	200-499 No (%)	>500 No(%)	*P* - value	<200 No(%)	200-499 No(%)	>500(%)	*P*- value	<200 No(%)	200-499 No(%)	>500 No(%)	*P* - value
*C. parvum*	132(44)	8(47.1)	11(52.4)	3(21.4)	0.171	36(61)	53(52.5)	21(23.9)	0.000	44(57.8)	65(52.8)	23(22.7)	0.000
*B. hominis*	75(25)	6(35.3)	5(23.8)	3(21.4)	0.63	13(22)	33(32.7)	15(17.1)	0.039	19(25.0)	38(30.8)	18(17.8)	0.080
*Microsporidium spp.*	64(21.3)	10(58.8)	4(19.1)	0(0)	0.006	23(39)	23(22.8)	4(4.6)	0.000	33(43.4)	27(21.9)	4(3.9)	0.000
*E. hystolytica*	22(7.3)	0(0)	4(19.1)	2(14.3)	0.175	1(1.7)	7(6.9)	8(9.1)	0.195	1(1.3)	12(9.7)	9(8.9)	0.065
*Isospora belli*	13(4.3)	1(5.9)	0(0)	0(0)	0.43	7(11.9)	5(5)	0(0)	0.003	8(10.5)	5(4.1)	0(0.0)	0.003
*C. cayetanensis*	11(3.6)	0(0)	2(9.5)	0(0)	0.29	4(6.8)	4(4)	1(1.1)	0.15	4(5.2)	6(4.8)	1(0.9)	0.211
*G. lambia*	10(3.3)	0(0)	1(4.8)	1(7.1)	0.731*	0(0)	4(4)	4(4.6)	0.307*	0(0.0)	5(4.1)	5(4.9)	0.162
*Lumbricoides*	13(4.3)	1(5.9)	0(0)	1(7.1)	0.509*	2(3.4)	3(3)	6(6.8)	0.421*	3(3.9)	3(2.4)	7(6.9)	0.255
Hookworm	8(2.7)	0(0)	0(0)	0(0)	-	0(0)	2(2)	6(6.8)	0.072*	0(0.0)	2(1.6)	6(5.9)	0.034*
*T. trichuria*	7(2.3)	0(0)	1(4.8)	1(7.1)	0.731*	1(1.7)	1(1)	3(3.4)	0.543*	1(1.3)	2(1.6)	4(3.9)	0.409*
*H. nana*	4(1.3)	0(0)	0(0)	1(7.1)	0.269*	0(0)	3(3)	0(0)	0.183*	0(0.0)	3(2.4)	1(0.9)	0.323*
*S. stercoralis*	2(0.7)	0(0)	0(0)	0(0)	-	2(3.4)	0(0)	0(0)	0.056*	2(2.6)	0(0.0)	0(0.0)	-
Total	361	26	28	12		89	138	68		115	168	78	

The number of pathogens exceeds the number of patients as multiple parasites were identified in some patients

*P value from Fisher’s exact test

### Association of intestinal parasite with diarrhea among pre-ART and on-ART HIV/AIDS patients

A total of, 103 (87.3 %) of the 118 participants who presented with diarrhea had intestinal parasites while 145 (79.7 %) of the 182 participants without diarrhea had intestinal parasites (Table 5). Overall, no significant association was observed between the presence of IPI and diarrhea (*p* = 0.08). No significant association was also observed between the prevalence of IPI and diarrhea among pre-ART (*p* = 0.083) and on-ART (*p* = 0.3) HIV patients.

**Table 5:** The occurrence of diarrhea among the study participants stratified by ART status and IPI

Diarrhea status	n	Overall		Pre-ART		On-ART	
		Parasites present No (%)	Parasites absent No (%)	Parasites present No (%)	Parasites absent No (%)	Parasites present No (%)	Parasites absent No (%)
Present	118	103(87.3)	15(12.7)	25 (96.2)	1(3.8)	78(84.8)	14(15.2)
Absent	182	145(79.7)	37(20.3)	21(80.8)	5(19.2)	124(79.5)	32(20.5)
Total	300	248(82.6)	52(17.3)	46(88.5)	6(11.5)	204(81.6)	46(18.4)
*χ* ^2^, *P*-value		*χ* ^2^ = 2.88, *p* = 0.08		*χ* ^2^ = 3.015, *p* = 0.083		*χ* ^2^ = 1.074, *p* = 0.300	

*χ* ^2^ = Chi square values for the association between diarrhea, parasites with Antiretroviral therapy

Species-specific analysis indicated that diarrhea was observed to be associated with infection with *Cryptosporidium parvum*, *Cyclospora cayetanensis*, and *Microsporidium spp* (*p* = 0.008, *p* = 0.028, *p* = 0.011 respectively) (Table 6). Among pre-ART HIV patients, *Cryptosporidium parvum* was observed to be significantly associated with diarrhea (*p* = 0.025) while no intestinal parasite was significantly associated with diarrhea among pre-ART HIV patients (Table 6).

**Table 6:** Association between intestinal parasite and Diarrhea

Parasites	n	Pre-ART			On-ART			Overall		
		Presence of Diarrhea No (%)	Absence of Diarrhea No (%)	*P*-value	Presence of Diarrhea No (%)	Absence of Diarrhea No (%)	*P*-value	Presence of Diarrhea No (%)	Absence of Diarrhea No (%)	*P*- values
*C. parvum*	132	15(57.7)	7(26.9)	0.025	48(52.2)	62(39.7)	0.057	63(53.4)	69(37.9)	0.008
*B. hominis*	75	7(26.9)	7(26.9)	1.000	15(16.3)	46(29.5)	0.020	22(18.6)	53(29.1)	0.041
*Microsporidium spp.*	64	9(34.6)	5(19.2)	0.211	25(27.2)	25(16)	0.035	34(28.8)	30(16.5)	0.011
*E. hystolytica*	22	1(3.9)	5(23.8)	0.083	3(3.2)	13(8.3)	0.116	4(3.4)	18(9.9)	0.035
*I. Belli*	13	1(3.9)	0(0)	0.313	7(7.6)	5(3.2)	0.118	8(6.8)	5(2.8)	0.094
*C. cayetanensis*	11	2(7.7)	0(0)	0.490*	6(6.5)	3(1.9)	0.081*	8(6.8)	3(1.7)	0.028*
*A. lumbricoides*	13	1(4)	1(4)	1.000	1(1.1)	10(6.4)	0.058	2(1.7)	11(6)	0.071
*G. lambia*	10	0(0)	2(7.7)	0.490*	4(4.4)	4(2.6)	0.474*	4(3.4)	6(3.4)	1.000*
*Hookworm*	8	0(0)	0(0)	1.000*	0(0)	8(5.1)	0.028*	0(0)	8(4.6)	0.024*
*T. trichuria*	7	0(0)	2(7.7)	0.490*	1(1.1)	4(2.6)	0.654*	1(0.9)	6(3.4)	0.252*
*H. nana*	4	0(0)	1(3,9)	1.000*	3(3.2)	0(0)	0.050*	3(2.5)	1(0.6)	0.304*
*S. stercoralis*	2	0(0)	0(0)	1.000*	2(2.2)	0(0)	0.137*	2(1.7)	0(0)	0.154*
*Total*	361	36	30		115	180		151	210	

The number of pathogens exceeds the number of patients as multiple parasites were identified in some patients

*P value from Fisher’s exact test

A highly significant association was observed between CD4^+^ T cell count and diarrhea (*P* = 0.000). The risk of diarrhea was observed to increase with decreasing CD4^+^ T cell count independent of parasitic infection and with HIV patients with counts below 200 cells/μl having excess risk compared to those with CD4^+^ T cell count above 500 cells/μl (COR = 10.21, *p* = 0.000).

## Discussion

Intestinal parasitic infections (IPI) are a major public health concern in HIV/AIDS patients in developing countries particularly in Sub-Saharan Africa where the highest burden of HIV is also recorded [4]. Antiretroviral therapy (ART) which is now widely available in the region has brought about a significant improvement in the lives of HIV-infected persons. Studies comparing the prevalence and associated factors of IPI among HIV/AIDS patients on-ART compared to pre-ART patients particularly in Sub-Saharan Africa are not readily available hence the need to investigate the prevalence of intestinal parasites in relation to the CD4^+^ T cell count and diarrheic status in HIV/AIDS patients before and during administration of ART.

In this study, there was no statistically significant difference (*p* = 0.128) between the prevalence (88 % vs. 81.6 %) of IPI in pre-ART compared to on-ART patients respectively which was in contrast to the study by Missaye and colleagues [18] in Ethiopia. A high prevalence of intestinal protozoa than intestinal helminthes (74.3 % vs. 11.3 %) was is in line with studies performed elsewhere [10, 18, 33, 34]. *Cryptosporidium parvum* was highly prevalent (44 %) in both pre-ART and on-ART HIV patients, which was in contrast to studies that have reported *Entamoeba histolytica/dispar* as the most prevalent intestinal parasite in HIV/AIDS patients [10, 11, 18], as well as those reporting *Blastocystis hominis* as the most prevalent intestinal parasite [33]. The prevalence of *Cryptosporidium parvum* observed in this study is very high compare to studies which have reported prevalence in the range of 2.2 to 15.8 % [1, 33, 35–38]. On the contrary this high prevalence of *Cryptosporidium* was in line with the study performed by Missaye and collleagues [18]. *Cryptosporidium parvum*, *Cyclospora cayetanensis*, *Isospora belli* and *Microsporidia* are often recognized as opportunistic parasites in HIV/AIDS patients because they tend to be present when the CD4^+^ T cell counts are below 200 cells/μl [9]. Parasite species was observed to be significantly associated with ART status which is contrary to the study by Missaye and colleagues [18] in which *Enatamoeba histolytica* was observed to be significantly higher in pre-ART patients.

In this study, IPI prevalence was significantly higher in patients with CD4^+^ T cell count below 200 cells/μl (*p* = 0.000) which is in line with studies published elsewhere [10, 11, 18]. Among the parasites isolated, only *Cryptosporidium parvum*, *Microsporidium spp*., *Isospora belli* and hookworm were observed to be significantly associated with CD4^+^ T cell count. These parasites with the exception of hookworm take advantage of the low CD4^+^ T thus causing infection in these patients. Cellular immunity is the major defense against intestinal parasitic infections. Therefore, the reduction in CD4^+^ T cell count by the HIV virus predisposes HIV-infected patients to these opportunistic intestinal parasitic infections. Other studies have also reported a significant association of CD4^+^ T cell with increase *Cryptosporidium parvum* [10, 18], and *Isospora belli* [10]. In this study, only *Microsporidium spp*. was significantly associated with low CD4^+^ T cell count among pre-ART HIV patients meanwhile *C. parvum*, *B. hominis*, *Microsporidium spp*. and *I. belli* were all significantly associated with low CD4^+^ T cell count in the on-ART group. This is contrary to the study by Missaye and colleagues [18] in which *Cryptosporidium parvum* was the only species observed to be significantly associated with CD4^+^ T cell count below 200 cells/μl in pre-ART patients.

Overall, no significant association was observed between IPI and diarrhea which is very much in line with the study by Assefa and colleagues [10] but contrary to the study by Teklemariam and colleagues [11] where a significant association was observed. The opportunistic parasites; *Crytosporidium parvum, Blastocystis hominis* and *Microsporidium spp*. were significantly associated with diarrhea. These parasites being intracellular disrupt epithelial cells resulting to fluid and electrolyte loss. Patients with CD4^+^ T cell count below 200 cells/μl were more likely to have diarrhea (COR = 10.21, *p* = 0.000) which is in line with studies performed elsewhere [10, 11]. This confirms that the weakened immunity predisposes HIV patients to infection with opportunistic parasites which worsen the prognosis hence the onset of diarrhea. Among pre-ART HIV patients, *C. parvum* was observed to be significantly associated with diarrhea meanwhile no intestinal parasite was observed to be associated with diarrhea in the on-ART group. This suggests that ART plays a role in preventing the development of diarrhea as a result of IPI in HIV/AIDS patients, which could be beneficial for a better health of these patients.

## Conclusion

The prevalence of IPI was significantly higher in HIV/AIDS patients with CD4^+^ T cell count below 200 cells/μl. *Microsporidium spp.* was significantly associated with low CD4^+^ T cell counts in pre-ART. Only *Cryptosporidium parvum* was significantly associated with diarrhea in pre-ART HIV patients with none in the on-ART group. This study clearly shows that low CD4^+^ T cell count appeared to be a risk factor for infection with intestinal parasites and the development of diarrhea in HIV/AIDS irrespective of the ART status. Regular screening and treatment for intestinal parasites and the strengthening of adherence to ART to increase the CD4^+^ T cell counts is of prime importance to improve the overall wellbeing of HIV/AIDS patients in this study area.

## Abbreviations

IPI: Intestinal Parasitic infection
ART: Antiretroviral Therapy
HIV: Human Immunodeficiency Virus
AIDS: Acquired Immunodeficiency Syndrome
CD: Cluster of Differentiation
